# Relationships between Reward Sensitivity, Risk-Taking and Family History of Alcoholism during an Interactive Competitive fMRI Task

**DOI:** 10.1371/journal.pone.0088188

**Published:** 2014-02-04

**Authors:** Haley L. Yarosh, Christopher J. Hyatt, Shashwath A. Meda, Rachel Jiantonio-Kelly, Marc N. Potenza, Michal Assaf, Godfrey D.Pearlson

**Affiliations:** 1 Olin Neuropsychiatry Research Center, Institute of Living at Hartford Hospital, Hartford, Connecticut, United States of America; 2 Department of Psychiatry, Yale University School of Medicine, New Haven, Connecticut, United States of America; 3 Department of Neurobiology, Yale University School of Medicine, New Haven, Connecticut, United States of America; 4 Child Study Center, Yale University School of Medicine, New Haven, Connecticut, United States of America; George Mason University, United States of America

## Abstract

**Background:**

Individuals with a positive family history for alcoholism (FHP) have shown differences from family-history-negative (FHN) individuals in the neural correlates of reward processing. FHP, compared to FHN individuals, demonstrate relatively diminished ventral striatal activation during anticipation of monetary rewards, and the degree of ventral striatal activation shows an inverse correlation with specific impulsivity measures in alcohol-dependent individuals. Rewards in socially interactive contexts relate importantly to addictive propensities, yet have not been examined with respect to how their neural underpinnings relate to impulsivity-related measures. Here we describe impulsivity measures in FHN and FHP individuals as they relate to a socially interactive functional magnetic resonance imaging (fMRI) task.

**Methods:**

Forty FHP and 29 FHN subjects without histories of Axis-I disorders completed a socially interactive Domino task during functional magnetic resonance imaging and completed self-report and behavioral impulsivity-related assessments.

**Results:**

FHP compared to FHN individuals showed higher scores (p = .004) on one impulsivity-related factor relating to both compulsivity (Padua Inventory) and reward/punishment sensitivity (Sensitivity to Punishment/Sensitivity to Reward Questionnaire). Multiple regression analysis within a reward-related network revealed a correlation between risk-taking (involving another impulsivity-related factor, the Balloon Analog Risk Task (BART)) and right ventral striatum activation under reward >punishment contrast (p<0.05 FWE corrected) in the social task.

**Conclusions:**

Behavioral risk-taking scores may be more closely associated with neural correlates of reward responsiveness in socially interactive contexts than are FH status or impulsivity-related self-report measures. These findings suggest that risk-taking assessments be examined further in socially interactive settings relevant to addictive behaviors.

## Introduction

Impulsivity is a complex, multi-faceted construct characterized by premature motor responses, decreased inhibition, abnormal reward sensitivity and risk-associated behaviors [1], [2]. Aspects of impulsivity-related behaviors are associated with neural responses to rewards and punishments [3]–[6]. Brain regions implicated in these processes include the amygdala [7], anterior cingulate cortex (ACC) [8], nucleus accumbens (NAcc), orbitofrontal cortex (OFC)[9]–[12], medial prefrontal cortex (mPFC) [13], [14], striatum [12], [15], [16], insula [4], [13], [17], [18], ventral tegmental area (VTA) [19], hippocampus [20], and subthalamic nucleus (STN) [21]. In particular, ventral striatal (VS) responses during processing of rewards and punishments, more specifically reward anticipation, may represent endophenotypes for disorders characterized by impaired impulse control such as alcoholism, pathological gambling, and binge eating disorder [6], [13], [22], [23].

Several hypotheses have been advanced to explain neural activity underlying reward processing as related to impulse control. The “reward deficiency hypothesis” suggests that individuals compensate for trait-like under-activation of neural-based reward response with risk-taking and impulsive behaviors [24]. This is consistent with observations of blunted VS responses during reward anticipation and lower availability of D2-like dopamine receptors in drug abuse/dependence [25]–[27].

The term “impulsivity” is sometimes used broadly and imprecisely [28]–[30]. Although multiple studies discuss ‘trait impulsivity’ or ‘impulsive behavior’, few have used multiple standard assessments in the same subjects to examine relationships between behavioral risk-taking, self-reported risk-taking, and social influences on these tendencies and behaviors. Furthermore, few studies investigate multiple impulsivity-related characteristics in a single clinical population. Using principal component analysis, we previously identified five impulsivity-related domains that comprise: 1) self-reported behavioral activation, 2) self-reported compulsivity and reward/punishment sensitivity, 3) self-reported impulsivity, 4) behavioral temporal discounting, and 5) behavioral risk-taking [12], [16], [18], [19], [29], [31], [32].

Lineages with heavy drug and alcohol use disorders (AUDs) show higher prevalence of impulsive behaviors, including those related to reward-processing differences [12], [30], [33]–[42]. Children of alcohol-dependent parents (family-history-positive (FHP) for alcoholism) are more likely to demonstrate under-controlled behavior, make impulsive errors, exhibit steeper delay discounting curves and perform more poorly on cognitive-control and decision-making measures compared to family-history-negative (FHN) peers [43]–[47]. They are also likely to initiate drug and alcohol use earlier [48]. While social environments and time of initial encounter with substances may influence substance-use behaviors, family history of alcoholism (FHA) is arguably the single greatest risk factor for developing substance-use disorders [49], [50]. Data suggest heritable factors underlie heightened impulsivity-related behaviors in alcohol abuse/dependence. FHP individuals may express increased hedonic and stimulatory responses to the effects of alcohol and related cues [51], [52]. Non-drinking children of alcoholic and drug-abusing parents show risk-taking and sensation-seeking attributes independent of alcohol and drug use [53]–[56].

In the current study, we investigated relationships between FHA, impulsivity-related domains and brain activations assessed using functional magnetic resonance imaging (fMRI) during performance of an interactive competitive Domino task that includes both risk-taking and rewards/punishments. Each round of the Domino task includes an outcome interval with a notification of reward/punishment. Impulsivity-related domains were examined using a factor analysis approach that we have employed previously [6], [29], [31], [42]. We aimed to investigate whether: a) impulsivity-related domains are associated with reward-related neural activity across all subjects, and b) FHA associated with impulsivity-related factor scores or fMRI reward response during the Domino task. We hypothesized that we would observe between-group differences (FHP/FHN) in reward-related activity in targeted regions of interest (ROIs): ventral and dorsal striatum [54], [57]. We also hypothesized that high scores on behavioral risk-taking would be associated with reward-related neural activity based on prior research showing that such brain activity correlated highly with behavioral risk-taking [58].

## Materials and Methods

### Ethics Statement

The experimental protocol and the study were approved by the Human Research Protections Program (HRPP) of the Hartford Hospital Institutional Review Board (IRB) and Yale University Institutional Review Board (HIC/HSC). The studies were carried out at Hartford Hospital Institute of Living. All study participants provided written informed consent after the study had been fully explained to them. Participants were paid for participating in the imaging study. Sixty-nine healthy individuals, 40 FHP and 29 FHN, were recruited by word-of-mouth, flyers, newspaper, online advertisement and drug abuse programs.

### Study Participants

Sixty-nine healthy individuals, 40 FHP and 29 FHN were recruited into two groups based on FHA using detailed interviews. These subjects include 7 FHP and 11 FHN individuals who participated in a prior study employing the MID task, and additionally included 33 FHP and 18 FHN subjects [6]. As previously, FHN was defined as having no first-degree relative with alcohol-abuse history; FHP subjects reported an alcohol-dependent father and alcohol abuse or dependence in one or more first-degree or second-degree relatives. Potential FHP subjects that have mothers with a lifetime history of alcohol abuse were excluded to avoid confounds related to fetal alcohol exposure. Subjects meeting neither group criteria (e.g., alcoholism in several second-degree, but no first-degree, relatives) were excluded. Exclusion criteria included current or past history of any Axis-I diagnosis, assessed by the Structured Clinical Interviewed for DSM-IV Axis-I Disorders (SCID), major physical illness, current or past history of central neurological disease or substance abuse or dependence, history of head trauma causing loss of consciousness>10 min, MRI contraindications, poor understanding of spoken/written English, auditory or visual impairments impeding test-taking, positive urine toxicology for commonly abused substances, positive pregnancy screen, and positive alcohol breath screen at the time of fMRI scanning [59]. Subjects were group-matched for age and sex as detailed in Table 1. Additionally, participants were asked to record on which days they drank, and how many drinks were consumed each day over a one-month period prior to their visit. An independent samples T-test reveals a significant difference between groups (p = .007, F = 8.270) on number of days participants drank during the month, but no difference between drinks per episode (p = .491, F = .481).

**Table 1:** Subject Demographics.

Demographic	FHN	FHP	Total	Chi-Square	p Value
Gender (M:F Ratio)	11∶18	14∶26	29∶40	0.063	0.80
Ethnicity (White/Hispanic/AfricanAmerican/Asian/Other)	27/0/1/0/1	36/1/2/0/1	63/1/3/0/2	1.654	0.80
Age, years (Mean± SD)	25.45±6.33	26.15±5.58	25.86±5.87		

M = male, F = female.

Demographics for FHN, FHP, and total sample used in the study.

### Domino Task

Subjects performed the Domino task described previously [16], [60], [61]. This two-player competitive game involves participant- and computer-generated responses, though players are told that they have a human opponent to mimic an interpersonal competitive interaction. This creates a social context for the participant. At the start of each game, 12 random Domino-like chips from a pool of 28are shown face up on the screen and assigned to the player. An opponent’s Domino chip (constant throughout the game) shows face-up on the top-corner of the board. Four undisclosed chips are assigned to a bank, leaving 11 remaining chips not used for the current game. The player’s goal is to dispose of all assigned chips before the game ends (4 min). Each assigned chip can either match the opponent’s (match one of the opponent chip’s numbers) or not. Playing a matching chip is considered a ‘safe’ move because it will not result in punishment. Non-matching chips are considered ‘risky’ moves or ‘bluffs’, since they can result in a gain of chips if challenged by the opponent. A player must occasionally bluff to win the game, since he/she will not always have a matching chip to play. Each game is played for a $10 reward bonus; losing a game results in no bonus.

**Figure 1:**
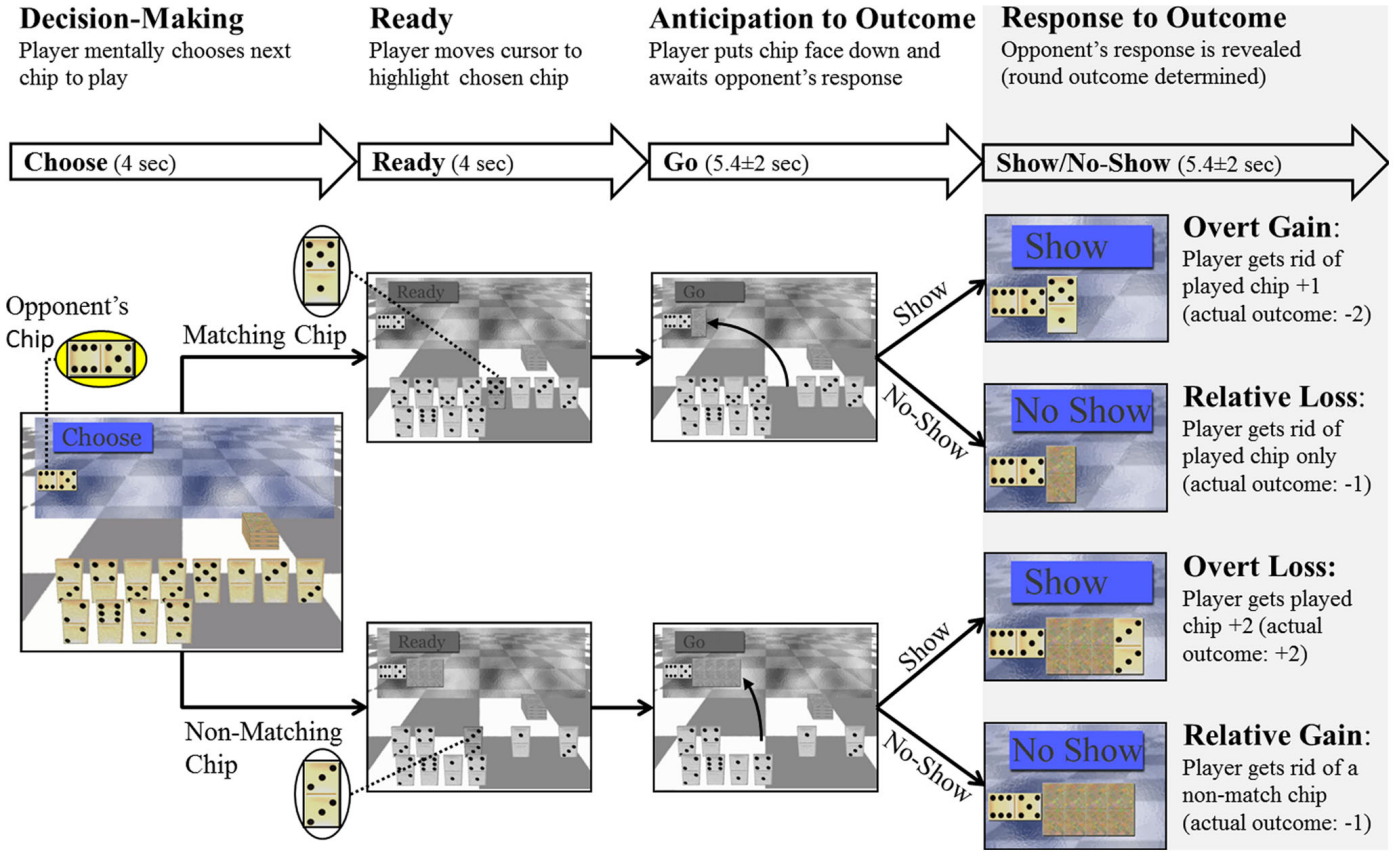
Domino game task. Domino Game Paradigm. The upper panel describes the 4 intervals that comprise each round of the game: Decision Making, Ready, Anticipation to Outcome and Response to Outcome. The latter is the main focus of this study, thus it is highlighted in gray. The duration of each interval and the command (i.e. event) that starts it are described in the bolded arrows below. The lower panel depicts the Domino Game sequence and corresponding consequences. At the beginning of each game the player (participant scanned) gets 12 playing chips and his/her goal is dispose of all 12 within 4 minutes. A constant opponent’s chip (in this example 6∶5, shown enlarged in the yellow ellipsoid) to which the player matches one chip in each round of the game, is displayed in the upper left corner of the screen throughout the game. Each round starts with the player instructed to decide what chip he/she will play next by the command ‘Choose’ (Decision-making interval). Then the player is instructed to move the cursor to this chip (Ready interval). The chip can either match the opponent’s (i.e. have one of the numbers match those on the opponent’s chip, upper row, 5∶1 in this example) or not (lower row: 3∶3; note that this is a later round in the same game). After placing the selected chip face down next to the opponent’s, he/she awaits the opponent’s response (Anticipation of Outcome interval). The opponent can either challenge the player’s choice (‘Show’) or not (‘No-Show’). Based on the player’s choice and the opponent’s response there are four possible consequences for each round (Response to Outcome interval): Show Match (overt gain); No-Show Match (relative loss, as the player could have been rewarded if challenged); Show Non-Match (overt loss) and No-Show Non-Match (relative gain, as the player could have been punished if challenged).

Subjects play two rounds of the game, each 15 min in duration, where the player decides which chip to play next and awaits the opponent’s response. The opponent can either challenge the player by asking him/her to reveal (turn face-up) the chosen chip or not challenge, allowing him/her to move on to the next round. Players are given visual and aural instructions throughout the round (Figure 1). (a) ‘Choose’ instructs the subject to mentally select a chip to be played next (4 sec). (b) ‘Ready’ instructs the subject to move a cursor (using his/her dominant hand) to the chosen chip (4 sec). (c) ‘Go’ instructs the player to put the chosen chip face down next to the opponent’s chip. The player then awaits the opponent’s response (3.4 sec, 5.4 sec or 7.4 sec) of either (d) ‘Show’ or ‘No-Show’. The former command exposes the player’s selected chip (revealing whether they played safe or bluffed), while the latter leaves it unexposed. The next round of the game starts by presenting the ‘Choose’ command.

Based on the players’ choice and opponent’s response, there are four possible outcomes/consequences. (1) **Show matching chip**: player rewarded by disposal of the selected chip and one additional random chip from the game board (*overt gain*). (2) **Show non-matching chip**: the players’ choice of non-matching chip is exposed, and they are punished by receiving back the selected chip plus two additional chips (*overt loss*). (3) **No-show of non-matching chip**: a choice of a non-matching chip remains unexposed, and only the selected chip is disposed of (*relative gain*). (4) **No-show of matching chip**: the choice of a matching chip is not exposed and only the selected matching chip is disposed of (*relative loss*).

Rounds continue until subjects win (by getting rid of all of their chips) or lose (240 sec pass or they receive all the chips from the bank and the board back).

### Impulsivity-Related Measures

We administered two behavioral tasks and five self-report questionnaires, used previously to generate factor scores in five domains [29]. The assessments included the: (A) Behavioral Inhibition System/Behavioral Activation System (BIS/BAS) scale [62] B) Barratt Impulsivity Scale (BIS-11) [63], (C) Sensitivity to Punishment/Sensitivity to Reward Questionnaire (SPSRQ) [64], (D) Sensation-Seeking Scale (Form V) [65], and (E)Padua Inventory [66]. The computerized tasks included the (F) Balloon Analog Risk Task [67]and (G) Experiential Discounting Task [68]. To calculate new z-scores for the current subjects, we used a component score coefficient matrix described previously [29]. This matrix generated scores for five factors: Factor 1- “self-reported behavioral activation” (A), Factor 2 - “Self-Reported Compulsivity and Reward/Punishment Sensitivity”(C+E), Factor 3 - “Self-Reported Impulsivity” (B+D), Factor 4 - “Behavioral Temporal Discounting” (A+G), Factor 5 - “Behavioral Risk-Taking” (F).

### Behavioral Data Analysis for Domino Task

After completing the Domino game, subjects were administered a 44-question Likert-scale (1–5) questionnaire to assess understanding and emotional response to the task. Four questions measured emotional responses to choice outcome including the following statements: Show match/overt gain: “I felt glad when a matching chip was challenged”, No-show non-match/relative gain: “I felt glad when a non-matching chip was not challenged”, Show non-match/overt loss: “I felt angry when a non-matching chip was challenged” or No-show match/relative loss: “I felt unhappy when a matching chip was not challenged”. Between-group t-tests for each question response were run to determine group differences in response to reward and loss outcome.

To characterize player’s choices during the game, a Risk-Taking Index was defined as the ratio of the number of times a player chose a non-matching chip to the total number of choices. This index represents an unbiased choice when equal to 0.5 (exactly half of the choices were non-matching choices), a biased choice for matching chips when smaller than 0.5 or for non-matching chips when greater than 0.5.All behavioral statistics were compiled using SPSS™ software (v19, SPSS Inc., Chicago, IL).

### Functional MRI Acquisition for Domino Task

Blood oxygenation level dependent (BOLD) data were collected with a T2*-weighted echo-planar-imaging (EPI) sequence (TR/TE = 1,860/27 msec, Flip angle = 70°, Field of view = 22 cm with a 64×64 acquisition matrix) using a Siemens Allegra 3 Tesla scanner. Thirty-six contiguous axial functional slices of 3 mm thickness with 1 mm gap were acquired, yielding 3.4×3.4×4.0 mm voxels. Overall, 492 images were acquired per run, including six ‘dummy’ images at the beginning to allow global image intensity to reach equilibrium, which were excluded from data analysis.

### Functional MRI Data Analysis for Domino Task

Preprocessing: Imaging data were analyzed using SPM8 (Wellcome Department of Imaging Neuroscience, London, UK). Each individual’s data set was realigned to the first ‘non-dummy’T2* image using the INRIAlign toolbox [69], spatially normalized to the Montreal-Neurological-Institute space [70] using an EPI template, and spatially smoothed with a 9 mm-isotropic-FWHM Gaussian kernel. A high-pass filter with 128 sec cutoff was used to correct for EPI signal low-frequency drift. Participants with >3.5 mm of movement in x,y, or z direction during the task were excluded, eliminating six FHP and two FHN from assessment, leaving 40 FHP and 29 FHN subjects (Table 1).

Events and regressors: Ten first-level (subject-level) regressors were from the four game intervals described above (Figure 1): (1) choose-match and choose-nonmatch from the ‘Decision-making’ interval; (2) ready from the ‘Ready’ interval; (3) pick-match and pick- nonmatch from the ‘Anticipation of Outcome’ interval; (4) show-match, show-nonmatch, noshow-match and noshow-nonmatch from the ‘Response to Outcome’ interval, each regressor corresponding to the four consequences; (5) and a miscellaneous regressor for events of non-interest including Go events and between-game events, during which the subject learned whether they won or lost the last game and then waited for the next game to begin. The miscellaneous regressor also included all events occurring during games of <1 min duration; these were not analyzed.

Data were analyzed using a general-linear-model (GLM) approach using SPM8. Regressors were modeled as boxcar functions convolved with the SPM8 canonical hemodynamic response function (HRF) and included HRF temporal derivatives. All six movement parameters (translation: x, y and z; rotation: pitch, roll and yaw) were included as covariates of no interest in the model.

Contrast images and individual statistical parametric maps were calculated with contrasts from the four ‘Response to Outcome’ interval regressors in a fixed-effects first-level analysis. These include the Reward contrast, a linear combination of overt and relative win outcomes compared to baseline, the Punishment contrast, a linear combination of overt and relative loss compared with an implicit baseline, and finally the Reward-Punishment difference contrast. Based on previous analyses [16], the last contrast was used to delineate a reward network based on a one-sample t-map of neural activation from all 69 subjects (random effects one-sample t–test) to create a study specific “Reward-mask” (p_FWE_<0.05, corrected at the whole-brain level, *k* = 10 voxels). We chose this number to conservatively detect extended clusters of activation rather than isolated voxels.

To assess between-group differences in activation related to response to reward, Reward-Punishment individual contrast maps were entered into a random-effects 2-sample t-test. A random-effects multiple-regression analysis evaluated the association between reward-related brain activations and impulsivity-related factor scores. These group analyses were masked with the Reward-mask and thresholded at p_FWE_<0.05, corrected for voxels within the Reward-mask,*k* = 10 voxels.

## Results

### Domino Information

Subjects completed two 15 min scanning sessions, where there were 81.1±4.62 and 79.9±4.72 ‘Response to Outcome’ events for each FHP and FHN subject respectively (group differences via t-test F = 3.32, df = 2,68, p = .081) and subjects won 22.0% of their games (group difference via t-test, F = 0.015, df = 1,67, p = .90). There was an average of 52.7% gain events for FHP group and 52.6% gain events for FHN subjects (F = .127, df = 2,68, p = .72).

### Domino Debriefing

As in our previous report [16], gain responses were more salient than loss statement responses (p = .029), but there were no significant differences between absolute and relative loss and gain statements, respectively. As such, we grouped relative and absolute game events for task-related neural analysis. There was no mean between-group difference for each of these four responses (Table 2).

**Table 2:** Subject Debriefing after Domino Game.

*Group*	*Overt Gain*	*Relative Gain*	*Overt Loss*	*Relative Loss*
*FHN*	4.10±1.04	3.72±1.31	2.62±1.20	3.28±1.16
*FHP*	4.48±0.88	4.15±1.17	2.70±1.32	3.65±1.17
χ^2^	5.66	3.75	1.18	3.35
*p*	0.23	0.44	0.88	0.50

FHN = family history negative, FHP = family history positive.

Domino debriefing scores as Likert scale responses (1–5) to statements regarding salience of gains and losses during game play.

### Domino Risk-taking Propensity

**Figure 2:**
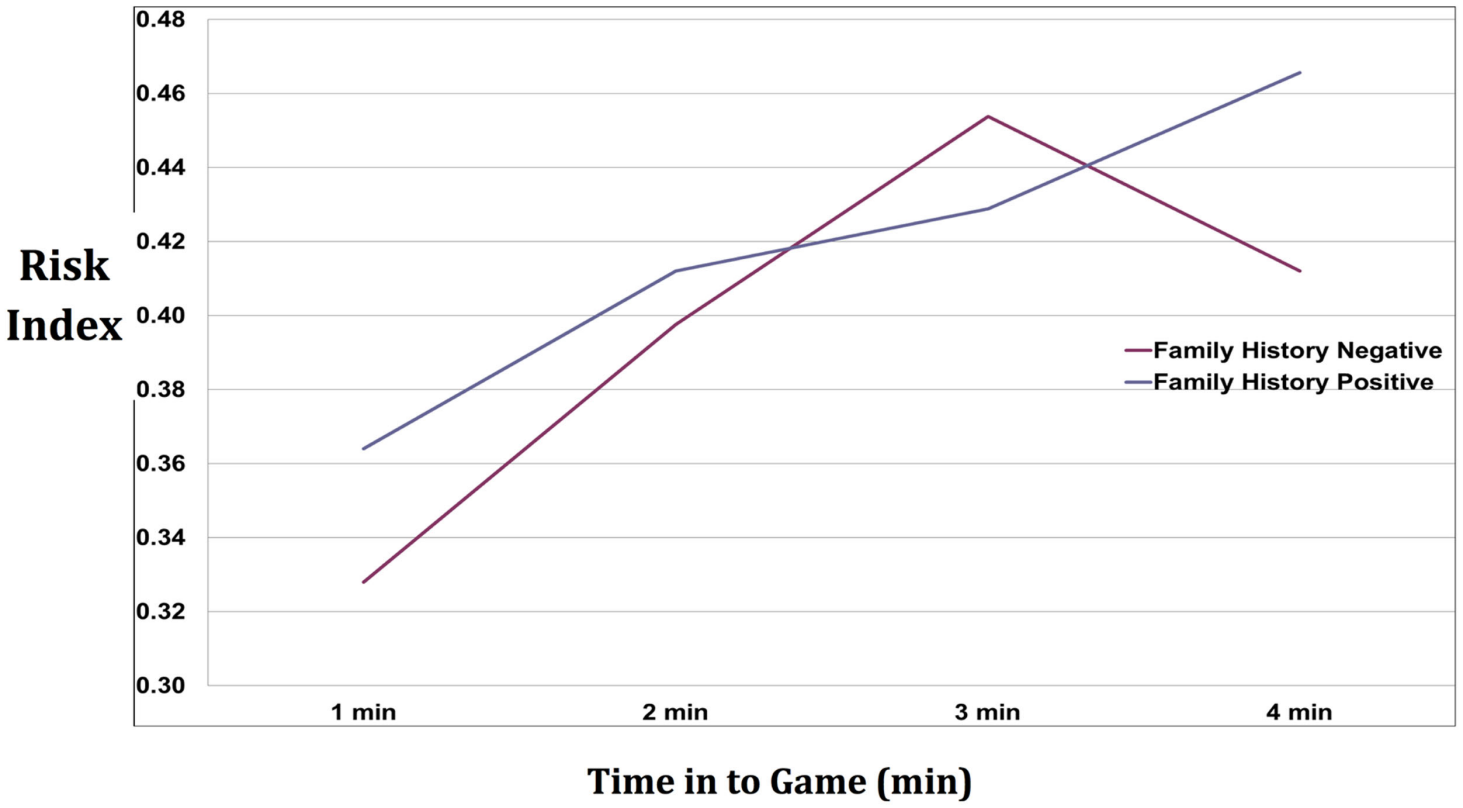
Risk indices by group (Family History Positive and Family History Negative) over time in to game. Risk Index was derived by dividing number of non-match choices by number of total choices when non-match and match choices were available to the player. FHP (a) and FHN (b) data are plotted by minute into game with standard error (averaged for all games for all subjects). No significant differences were found between groups. There was a significant main effect of time across groups (F = 9.58, df = 3, 201, p<.0001).

A Risk-Taking Index was generated by measuring tendency to ‘bluff’ over time, and a score was generated for each of four 1 min intervals per game. A two-way ANOVA (family-history-by-time-elapsed-into-game) revealed no effect of group (F = .52, df = 1,67, p = .47) and no group-by-time interaction. As we reported previously [16], there was a significant main effect of time for both groups (F = 9.58, df = 3, 201, p<.0001), indicating that players bluffed more often as the game progressed (Figure 2).

### Impulsivity-related Constructs

Independent-samples t-test of impulsivity-related factor scores by group revealed significant differences between groups for factor 2 (Self-reported Compulsivity and Reward-Punishment Sensitivity; F = .35, df = 1,67, p = .004), where FHP subjects had higher factor-2 scores. There was no interaction effect with or significant difference by group with factors 1, 3, 4 or 5.

### fMRI Analyses

**Figure 3:**
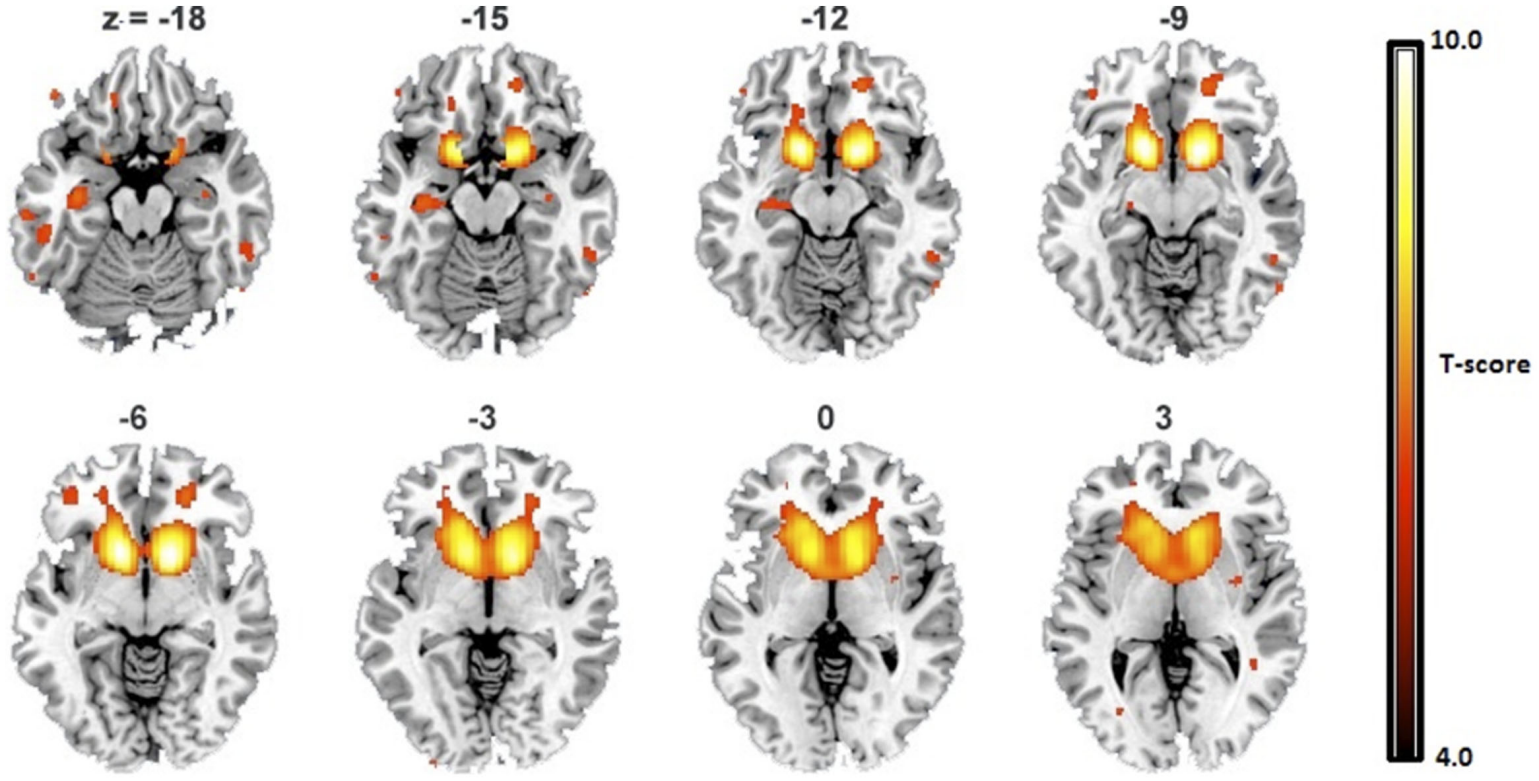
Reward Network. One-sample t-maps of the Reward-Punishment contrast for all subjects (*n* = 69) were used to construct the “reward network”. The threshold was set at p<0.05 FWE whole-brain corrected. Axial slices are depicted from z = −18 mm to z = +3 mm.

The Reward-mask obtained from contrasting reward and punishment events in the Response-to-Outcome interval included the caudate, putamen and ventral striatum (p_FWE_<0.05, k = 10; Figure 3). The Reward-mask was applied as an explicit mask to two-sample t-tests, but no statistically significant differences were observed between FHP and FHN subjects.

**Figure 4:**
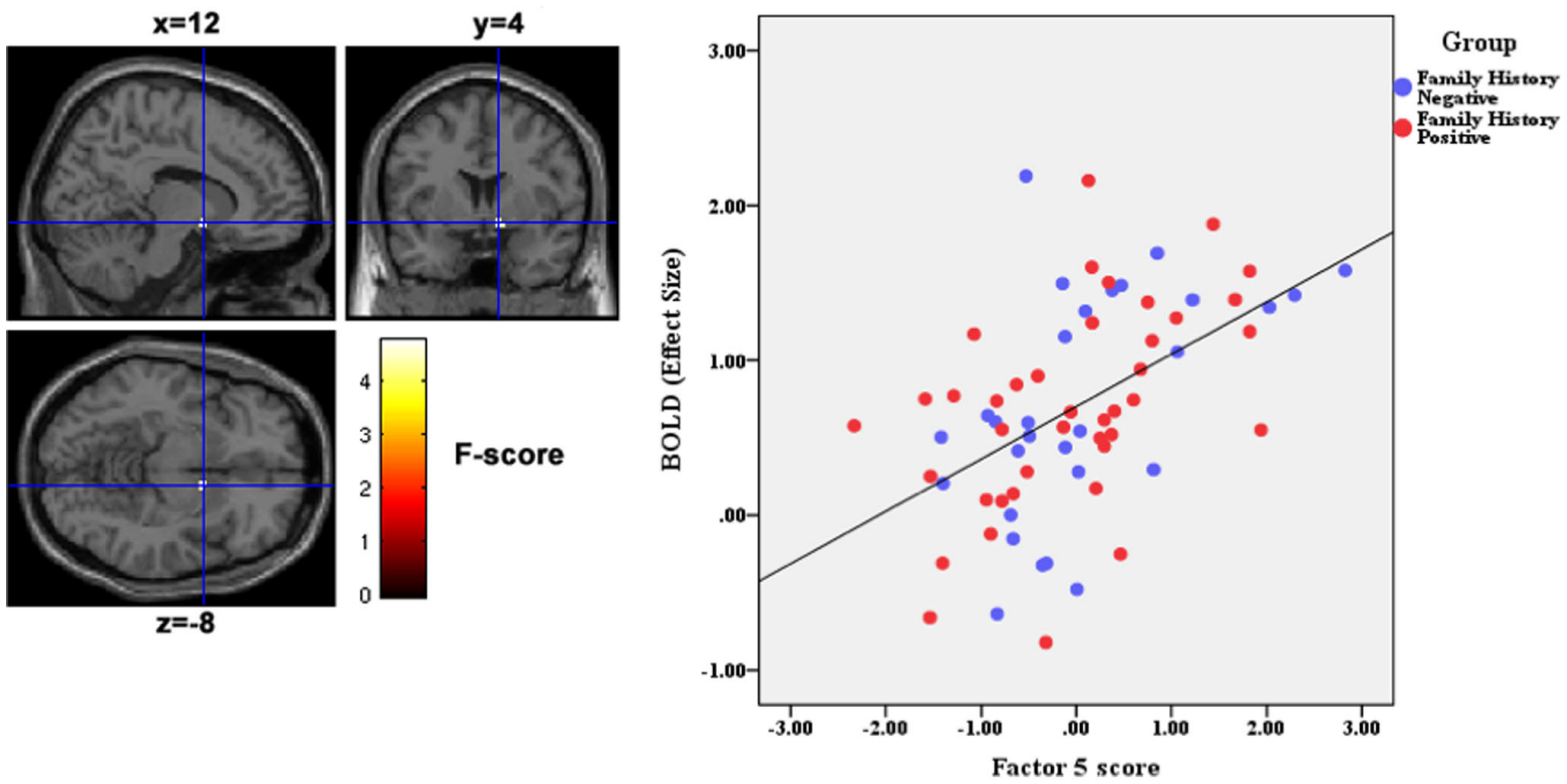
Correlation of Impulsivity-related Factor 5 (Behavorial Risk-Taking) scores with neural activation under the reward-punishment contrast across all subjects. **A**. Threshold was set at p<0.05 FWE for voxels within the reward network mask. Statistical parametric F-maps (sagittal, coronal and axial) of the Reward-Punishment contrast for multiple regression against Factor 5 - “Behavioral Risk-Taking”. Crosshairs overlaid on brain slices are at the voxel of peak correlation (MNI coordinates: x,y,z = 12,4,–8). Bottom panel depicts individual factor scores vs. individual peak cluster activation at this voxel. **B**. Linear regression plot at the voxel of peak correlation between reward-punishment BOLD activity in the right ventral caudate and Factor-5 score for each subject.

A multiple-regression analysis within the Reward-mask revealed a positive correlation across all subjects between impulsivity-related factor-5 scores and neural activity under the Reward-Punishment contrast in right ventral caudate(r^2^ = .26, T = 4.75, df = 63, p_FWE_ = .006 at coordinates x,y,z = 12,4,–8) (Figure 4).

## Discussion

Using a previously described interactive, competitive fMRI Domino task [16], [60], [61], we observed no differences in activation related to FHA in response to reward, focusing on a pre-defined, extended reward/motivation network. However, across all subjects, we observed a significant correlation between right ventral caudate activation to reward (assessed with reward>punishment contrast)and an empirically derived, impulsivity-related factor, “Behavioral Risk-Taking” (involving BART average adjusted number of pumps). In addition, impulsivity-related Factor 2 (Padua-Inventory and SPSRQ scores) comprising self-rated compulsivity and reward-punishment sensitivity, was significantly higher in FHP subjects, but not associated with regional task-related BOLD activation. This component has been associated with alcohol-use measures in a similar population [6]and related to insular/inferior-frontal-gyral activation during response inhibition [18]. Factor 2 also distinguished current cocaine users from both former cocaine users and healthy controls [16], [19], [71].

Based on previous research [60], [61], we expected that the Domino task would demonstrate FHA-related differences in neural reward responses in regions including the mesocorticolimbic circuit [72], [73], though the magnitude and direction of the association between impulsivity-related characteristics and these ROIs have been seemingly conflicting in previous studies. For example, during a Monetary Incentive Delay (MID) task performance in a subset of the same subject population, FHP versus FHN participants showed blunted activation in ventral striatum, OFC and insula during reward anticipation, and caudate hyperactivation during potential monetary reward outcome [6]. Ventral striatal hypoactivation during reward anticipation was also seen with an MID task in adolescents ages 18–22 years with AUD diagnoses in one or more parents [54], pathological gamblers [13], [74], adults with inattentive attention-deficit/hyperactivity disorder (ADHD) [75] detoxified male alcoholics [22], [27], binge-eating adults [23], and adolescent smokers [27]. Monetary rewards in the Domino task are not revealed to subjects until all games end. In contrast, the MID task reveals payment after each trial, which may be more participant-salient and account for group differences. Alternate possibilities include the socially competitive aspects of reward in the Domino but not MID task. The Domino Task is played in a social context, where participants are told that they have a human opponent. Neural activity related to reward and punishment outcomes may have both social and monetary influences; thus, the two tasks assess different aspects of these domains.

Both the VS and a functionally connected brain region, the ventromedial prefrontal cortex, are frequently implicated in reward-processing networks including tasks assessing decision-making under risk [76], [77]. We saw no hypoactivity in these regions as predicted by the reward-deficiency hypothesis. We did, however, see greater VS activation related to higher behavioral risk-taking (Factor 5) scores, possibly more consistent with a model of enhanced pro-motivational drive and diminished cognitive control. These findings might explain reward saliency/processing in at-risk individuals, as measured by risk-taking rather than FHA. The positive correlation with risk-taking differs from the inverse correlation seen with self-reported impulsivity and VS activation during reward anticipation during MID performance in individuals with alcohol dependence or pathological gambling [13]. The extent to which this represents differences in subject groups, tasks (including social and/or competitive aspects), risk-taking versus self-reported impulsivity or other factors warrants additional study.

One interpretation of our data is that individuals with greater BART risk-taking scores display activity in cognitive control and reward regions that is more typical of adolescence, a time when reward-seeking and impulsive behavior is maximal. Impaired adult reward response may be associated with atypical development of reward circuitry[2], [38], [78]–[80]. Adolescents engage VS more robustly than both children and adults during reward anticipation [56], [57] and receipt [55], [81]. However, some publications suggest the opposite is true in adolescents [57]. While this interpretation is speculative, it suggests that some impulsivity-related features might be based in persistently immature mesocorticolimbic circuitry.

FHP versus FHN subjects scored higher on impulsivity-related factor-2. This same factor was inversely correlated with VS BOLD response during the MID anticipation phase in our previous work investigating FHA, highlighting differences in neural responsiveness between reward anticipation and outcome presentation and the different task contexts [6]. In current and former cocaine-dependent subjects, impulsivity-related factor-2 scores correlated with right dorsal caudate BOLD signal under a reward>punishment contrast. Such factor analytically derived impulsivity-related measures are useful in clarifying relationships with brain biology. Additionally, these measures highlight neurobiological differences across at-risk and addicted populations.

A previous study of young adolescents with alcoholism vulnerability also reported no differences in reward-related neural responsiveness, but observed differences in sensation-seeking scores [57]. Impulsive behavior likely subsumes cognitive and motor control, in addition to reward sensitivity, and these neurobiological underpinnings should be parsed when considering the genetic and social influences and/or correlates of AUDs [82], analogous to animal models [83], [84]. VS is a germane neural region as it receives input from cortico-limbic regions involved in cognitive control [85]. The current data contribute to a growing literature on aspects of impulsivity-related constructs and FHA, a characteristic representing a possible risk factor for various disorders, including alcoholism, substance abuse, ADHD, binge-eating disorder, pathological gambling, and bipolar disorder.

We recognize study limitations. FHA relies on self-report data from individuals for themselves and family members. We also did not record data on history of subject smoking status, which could influence brain activation patterns. Our FHP subjects have passed early vulnerability ages for substance abuse and therefore may show more similar brain activation patterns to FHN subjects than other FHP individuals with substance-use disorders. As an interpersonal game, the Domino task includes neural activation during reward and punishment events subsuming both social and monetary elements. The ventral caudate has been linked with dysfunctional reward processing in other studies of disorders characterized by poor impulse control; these neural differences may be attributable to variations in motivational drive or cognitive control that should be examined directly in future research.

### Conclusions

Impulsivity is a broad trait with subfeatures that are not clearly distinguished by current standardized measurements. In this study, we demonstrate that FHP individuals exhibit risk-taking behavior that is different from FHN individuals by one impulsivity component. Also, behavioral risk-taking scores correlate with neural activity during reward responsiveness in the VS during an interactive competitive fMRI task in all subjects. This is a brain region that shows consistent association with impulsivity measures in previous tasks, and across several disorders. These findings show that impulsivity components may better highlight differences in disease states than a single measure.
